# Supplementary material for: Spatio-Temporal Variation in the Exceedance of Enterococci in Lake Burley Griffin: An Analysis of 16 Years’ Recreational Water Quality Monitoring Data
Source: Int J Environ Res Public Health. 2024 May 1;21(5):579. doi: 10.3390/ijerph21050579 (PMC11121496; doi:10.3390/ijerph21050579)
Supplement: Supplementary file 1 [file ijerph-21-00579-s001.zip › ijerph-2903543-supplementary.pdf]

Article

Spatio-Temporal Variation in the Exceedance of Enterococci in Lake Burley Griffin: An Analysis of 16 Years' Recreational Water Quality Monitoring Data

Ripon Kumar Adhikary [1,2,*], Danswell Starrs [3,4], David Wright [5], Barry Croke [6], Kathryn Glass [1] and Aparna Lal [1]

1 National Centre for Epidemiology and Population Health, Australian National University, Canberra 2601, Australia; kathryn.glass@anu.edu.au (K.G.); aparna.lal@anu.edu.au (A.L.)
2 Department of Fisheries and Marine Bioscience, Jashore University of Science and Technology, Jashore 7408, Bangladesh
3 Environment, Planning and Sustainable Development Directorate, ACT Government, Canberra 2601, Australia; danswell.starrs@act.gov.au
4 Research School of Biology, Australian National University, Canberra 2601, Australia
5 Lake and Dam, National Capital Authority, Canberra 2601, Australia; david.wright@nca.gov.au
6 Institute for Water Futures, Mathematical Sciences Institute and Fenner School of Environment and Society, Australian National University, Canberra 2601, Australia; barry.croke@anu.edu.au
* Correspondence: ripon.adhikary@anu.edu.au

Supplementary Materials:

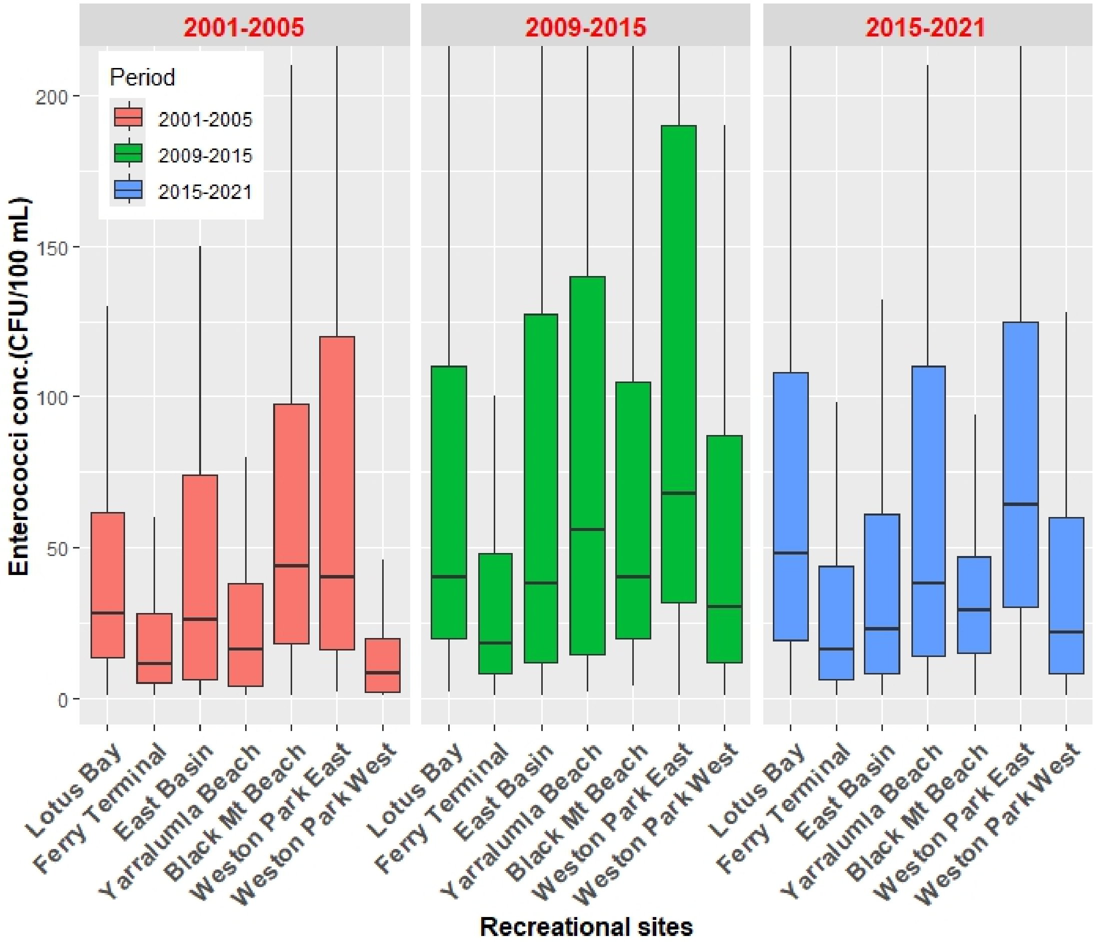

Figure S1. Percentile distribution of enterococci concentration in seven recreational sites over three periods (2001-2005, 2009-2015, 2015-2021)

Table S1. Descriptive statistics of study variables showing percentiles and p-values.

Variables	25th percentile	50th percentile (Median)	75th percentile	*p-value
Sites				
Lotus Bay	18	40	100	< 0.001
Ferry Terminal	6	14	40.5	
East Basin	10	26	82	
Yarralumla Beach	10	33	111.5	
Black Mountain Beach	17	34	80.5	
Weston Park East	24.5	60	157.5	
Weston Park West	6	20	60	
Months				
October	6	16	43.75	< 0.001
November	13.25	40	121.5	
December	16	40	100	
January	14	36	96	
February	16	40	101.5	
March	10	26	64	
April	10	22	54.5	
Years				
2001-2002	9	28	97	< 0.001
2002-2003	6	22	52	
2003-2004	6	16	49.5	
2004-2005	6	14	39.5	
2009-2010	16	32	76	
2010-2011	20	60	210	
2011-2012	16	46	142.5	
2012-2013	12	30	85	
2013-2014	12	36	120	
2014-2015	12	34	98	
2015-2016	12	30	78	
2016-2017	19	46	82	
2017-2018	22	48	102	
2018-2019	6	17	46.5	
2019-2020	6	18.5	83.5	
2020-2021	20	37	93.5	
Primary use of site				
Swimming activities	17	40	110	< 0.001
Non swimming activities	8	24	70	

*Kruskal-Wallis H test.

Table S2. Distribution of faecal indicator bacteria (measured by enterococci concentration) exceedance by frequency (n) and percentage (%) across sites, months, years, periods, and primary use of Lake Burley Griffin sites.

**Variables**	**Non-exceedance**		**Exceedance**		**Total**	***p*-value**
	**n**	**%**	**n**	**%**	**N**	
Sites						
Lotus Bay	367	87.59	52	12.41	419	
Ferry Terminal	396	94.74	22	5.26	418	
East Basin	366	87.14	54	12.86	420	
Yarralumla Beach	361	86.78	55	13.22	416	
Black Mountain Beach	377	90.41	40	9.59	417	
Weston Park East	339	81.49	77	18.51	416	
Weston Park West	392	93.78	26	6.22	418	
Months						
October	361	92.09	31	7.91	392	<0.001
November	373	83.26	75	16.74	448	
December	415	88.30	55	11.70	470	
January	422	88.47	55	11.53	477	
February	363	86.43	57	13.57	420	
March	419	92.09	36	7.91	455	
April	245	93.51	17	6.49	262	
Years						
2001-2002	159	84.57	29	15.43	188	<0.001
2002-2003	185	94.39	11	5.61	196	
2003-2004	180	95.24	9	4.76	189	
2004-2005	102	91.07	10	8.93	112	
2009-2010	111	93.28	8	6.72	119	
2010-2011	151	74.38	52	25.62	203	
2011-2012	174	82.86	36	17.14	210	
2012-2013	168	88.89	21	11.11	189	
2013-2014	161	87.50	23	12.50	184	
2014-2015	165	89.19	20	10.81	185	
2015-2016	183	93.37	13	6.63	196	
2016-2017	169	89.42	20	10.58	189	
2017-2018	175	89.29	21	10.71	196	
2018-2019	178	94.18	11	5.82	189	
2019-2020	168	88.42	22	11.58	190	
2020-2021	169	89.42	20	10.58	189	
Periods						
2001-2005	626	91.39	59	8.61	685	<0.001
2009-2015	930	85.32	160	14.68	1090	
2015-2021	1042	90.69	107	9.31	1149	
Primary use of site						
Swimming sites	1077	86.23	172	13.77	1249	<0.001
Non swimming sites	1521	90.81	154	9.19	1675	

*Pearson Chi-square test

Table S3. Frequency of enterococci concentration exceeding alert-level thresholds (N, Enterococci CFU per 100 mL > 200) and percentage of samples across seven recreational sites over three time periods.

Site name	2001-2005 Exceedance		2009-2015 Exceedance		2015-2021 Exceedance	
	N	%	N	%	N	%
Lotus Bay	7	7.14	25	15.92	20	12.2
Ferry Terminal	5	5.1	13	8.39	4	2.42
East Basin	14	14.29	25	15.82	15	9.15
Yarralumla Beach*	6	6.12	24	15.48	25	15.34
Black Mt. Beach*	11	11.22	22	14.19	7	4.27
Weston Park East*	14	14.43	36	23.23	27	16.46
Weston Park West	2	2.04	15	9.68	9	5.45
Total	59	8.61	160	14.68	107	9.31

*indicates designated swimming beach.

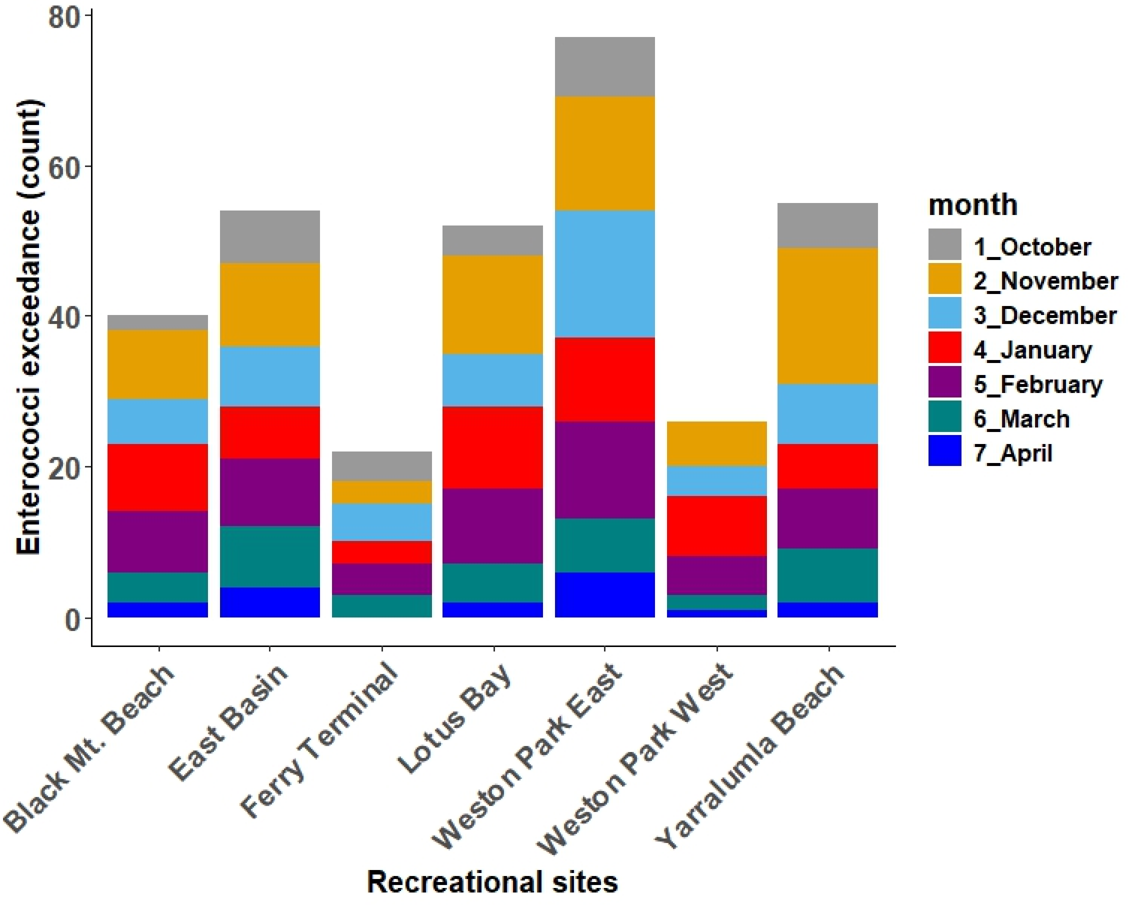

Figure S2. Frequency of enterococci concentration exceeding alert-level thresholds (N, Enterococci CFU per 100 mL > 200) across seven sites over seven recreational summer months.
